# Putative Origins of Cell-Free DNA in Humans: A Review of Active and Passive Nucleic Acid Release Mechanisms

**DOI:** 10.3390/ijms21218062

**Published:** 2020-10-29

**Authors:** Stefan Grabuschnig, Abel Jacobus Bronkhorst, Stefan Holdenrieder, Ingund Rosales Rodriguez, Klaus Peter Schliep, Daniel Schwendenwein, Vida Ungerer, Christoph Wilhelm Sensen

**Affiliations:** 1 Institute of Computational Biotechnology, Graz University of Technology, Petersgasse 14(V), 8010 Graz, Austria; stefan.grabuschnig@tugraz.at (S.G.); klaus.schliep@tugraz.at (K.P.S.); 2 Institute for Laboratory Medicine, German Heart Centre, Technical University Munich, Lazarettstraße 36, 80636 Munich, Germany; bronkhorst@dhm.mhn.de (A.J.B.); holdenrieder@dhm.mhn.de (S.H.); ungerer@dhm.mhn.de (V.U.); 3 CNA Diagnostics GmbH, Parkring 18, 8074 Grambach, Austria; ingund@cnadiagnostics.com (I.R.R.); daniel@cnadiagnostics.com (D.S.); 4 BioTechMed Graz, Mozartgasse 12/II, 8010 Graz, Austria

**Keywords:** cell-free DNA, circulating DNA, liquid biopsy, circulating tumor DNA, active release of cfDNA, passive release of cfDNA, origins of cfDNA

## Abstract

Through various pathways of cell death, degradation, and regulated extrusion, partial or complete genomes of various origins (e.g., host cells, fetal cells, and infiltrating viruses and microbes) are continuously shed into human body fluids in the form of segmented cell-free DNA (cfDNA) molecules. While the genetic complexity of total cfDNA is vast, the development of progressively efficient extraction, high-throughput sequencing, characterization via bioinformatics procedures, and detection have resulted in increasingly accurate partitioning and profiling of cfDNA subtypes. Not surprisingly, cfDNA analysis is emerging as a powerful clinical tool in many branches of medicine. In addition, the low invasiveness of longitudinal cfDNA sampling provides unprecedented access to study temporal genomic changes in a variety of contexts. However, the genetic diversity of cfDNA is also a great source of ambiguity and poses significant experimental and analytical challenges. For example, the cfDNA population in the bloodstream is heterogeneous and also fluctuates dynamically, differs between individuals, and exhibits numerous overlapping features despite often originating from different sources and processes. Therefore, a deeper understanding of the determining variables that impact the properties of cfDNA is crucial, however, thus far, is largely lacking. In this work we review recent and historical research on active vs. passive release mechanisms and estimate the significance and extent of their contribution to the composition of cfDNA.

## 1. Introduction

Cell-free DNA (cfDNA) is a fraction of circulating nucleic acids (CNAs), which was discovered and first described by Mandel and Metais, in 1948 [1]. The term encompasses all kinds of extracellular DNA molecules found in serum or plasma and other body fluids [2] of vertebrates and includes genomic and mitochondrial host DNA [3,4], as well as foreign DNA [5,6], for example, of bacterial or viral origin. The cfDNA molecules occur predominantly in the form of double-stranded DNA (dsDNA) [7] and are mostly of small size, ranging between 100 and 200 base pairs (bp) [8]. Larger fragments, even in the range of several kilobase pairs (Kbp) have also been reported [9,10,11].

In 1973, Leon et al. showed that cancer patients exhibited elevated levels of cfDNA in serum [12]. For this purpose, the group developed and performed a radioimmunoassay, where ***^125^***I-Iododeoxyuridine labelled DNA was detected via antibodies from a lupus erythematosus patient. Similar results had already been described by Tan et al., in 1966, [13] for systemic lupus erythematosus, and even as early as 1948, Mandel and Metais [1] showed that cfDNA levels were elevated in several disease conditions. Although the cfDNA concentrations in the serum declined in correlation with improved clinical conditions after treatment, Leon et al. were initially skeptical about the potential diagnostic value of cfDNA, because approximately half of the cancer patients had serum cfDNA concentrations that were within the same low range as found in healthy subjects. However, the group kept pursuing the idea of a cfDNA-based assay for benign and malignant gastrointestinal diseases [14] due to their discovery that high serum cfDNA levels were associated with other pathological conditions, such as rheumatoid arthritis [15] and pulmonary embolism [16].

Since then, a wide range of diagnostic approaches, collectively referred to as “liquid biopsies”, has been developed [17,18]. While cancer-related approaches have often targeted circulating tumor DNA (ctDNA), carrying cancer-specific genetic alterations [19,20], other strategies have involved length profiling of cfDNA molecules [10] or screening for epigenetic modifications [21], which were specific for various malignancies [22]. Another well-established application for cfDNA-based liquid biopsies is the cell-free foetal DNA (cffDNA)-based prenatal test for the detection of Down syndrome and other trisomies in maternal blood [23,24,25,26].

**Figure 1:**
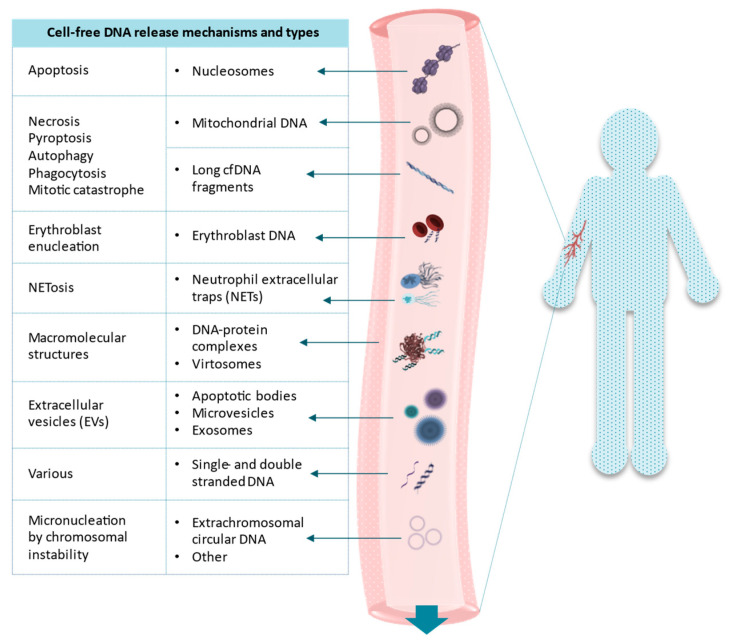
Different forms of cell-free DNA (cfDNA) in the human circulatory system. The biological and structural characteristics of the aggregate cfDNA population in a typical biospecimen is highly heterogeneous. While some overlap is common (see the main text), different cellular sources and mechanisms of origin often result in the production of uniquely distinct forms of cfDNA. Exhaustive stratification of the different cfDNA subtypes and an improved understanding of the factors which might modulate these characteristics of cfDNA are vital steps toward understanding the biological role(s) of cfDNA, as well as an expansion of their clinical utility.

Altogether, a large number of studies are currently focused on the diagnostic capabilities of cfDNA [17,18,22], while the characterization of the molecular mechanisms [27] underlying their release and biology remains largely neglected. Therefore, the purpose of this review is to outline the mechanisms of cfDNA release into the bloodstream and the biological properties of cfDNA molecules, as known thus far (Figure 1).

## 2. Apoptosis and Necrosis

For almost 20 years, apoptosis has been considered to be the primary source of circulating cfDNA in both healthy and diseased individuals [28,29,30], taking into account that about 50 to 70 billion cells perish via programmed cell death in the human body every day [31]. The maintenance of the homoeostatic balance and a steady turnover of cells are continuous tasks in multicellular organisms, as an imbalance between the formation of new cells and the removal of abnormal/blemished or superfluous cells can result in significant complications. Processes, such as oxidative stress and DNA damage, can trigger an imbalance in the cellular homeostasis, leading to the initiation of the intrinsic (mitochondrial) apoptotic pathway [32], which is controlled and carried out by the pro-apoptotic and pro-survival members of the BCL-2 family of proteins [33].

In contrast, the extrinsic apoptotic pathway is activated by the binding of extracellular signals (“death factors”) to their respective receptors (“death receptor”) [32] or, provided that specific conditions are met, to pattern recognition receptors (PRR) [34]. As different as the two pathways are, they both result in activation of a caspase-dependent proteolytic cascade, which is executed by initiator caspases (caspase-8 and -9) and, subsequently, executioner caspases (caspase-3, -6, and -7), which leads to inhibition of the inflammatory response [35], enzymatic degradation of cell components, DNA fragmentation, translocation of phosphatidylserine to the cell surface, formation of membrane blebs [36], and finally, packaging and release of extracellular vesicles (EVs) from apoptotic cells [37]. Inhibited or excessive apoptosis levels are connected to several medical conditions, including cancer, autoimmune, and neurodegenerative diseases [33].

The process of apoptotic nucleosomal DNA fragmentation starts with the concerted action of caspase-activated DNase (CAD), which is also termed DNA fragmentation factor subunit beta (DFFB), and DNaseI L-3 (or DNase γ), which translocates from the endoplasmatic reticulum to the nucleus when apoptosis has been initiated [38,39]. This DNase is involved in the degradation of intra- and extracellular DNA [40]. Since CAD is an endonuclease, lacking exonuclease activity, it cleaves the dsDNA of inter-nucleosomal linker regions [41,42], which is mirrored in nucleosomal footprints [43]. Extracellular DNaseI, which cleaves naked DNA that is not densely organized or protein-associated, is believed to be implicated in the “trimming” of cfDNA fragments to less than 150 bp in size [44] among other functions [40,45]. Watanabe and colleagues showed, in mouse experiments, that the deletion of CAD produced a single 180 bp DNA band, whereas the deletion of both CAD and DNaseI L-3 resulted in a decrease in cfDNA concentration and generation of cfDNA fragments of varying sizes (shorter or longer than mono-nucleosomal fragments and multiples thereof). A double deletion mutant, combining CAD and DNaseI L-3 deficiency, led to the complete absence of cfDNA [46]. Apart from DNaseI, further cfDNA degradation is executed partially in the bloodstream, for example, by plasma factor VII activating protease (FSAP) [47], which increases the accessibility of the DNA and Factor H [48], or by the liver, spleen, or kidney, as well as by affiliation with other blood constituents [49,50,51,52].

Apoptotic EVs are the main instrument to facilitate apoptotic cell clearance and to communicate with their environment, thereby initiating or inhibiting immune responses [37]. They comprise apoptotic bodies [53], apoptotic microvesicles, apoptotic exosomes [54], and apoptotic exosome-like particles [55]. Apoptotic bodies include proteins, lipids, (mi)RNA, and DNA [56,57,58,59]. Their production is believed to occur in a highly regimented manner [60,61]. According to Fernando et al. [62], more than 90% of cfDNA is related to exosomes, being located either on the surface of the vesicles or within the vesicle lumen [63].

Necrosis, i.e., accidental cell death, is a faster process than apoptosis which also contributes, in some instances, to the pool of cfDNA, mainly in the form of larger fragments (>1000 bp), but also short fragments, that are attributed to partial plasma nuclease cleavage of larger circulating fragments [64,65,66,67].

## 3. Erythroblast Enucleation

Roughly two million red blood cells (RBCs) with a half-life of approximately 115 to 120 days [68,69,70] mature every second in healthy adults [70,71]. Their complex biogenesis, starting from multipotent hematopoietic stem cells [71,72], involves several intermediate stages and occurs mostly in bone marrow, but also in the spleen and liver [71,73]. While nuclei of vertebrate RBCs become highly condensed, pyknotic, and transcriptionally inactive during maturation in general [69], mature mammalian erythrocytes even lack a nucleus [70,71,72]. This was discovered in 1875 by Gulliver [74] and was thought to enable higher hemoglobin levels in the blood [69,72]. The process of enucleation (formerly also called denucleation), which was visualized via electron microscopy by Simpson in 1967 [75], occurred at the stage of orthochromatic erythroblasts at the erythroblastic islands [70,71,73], which were discovered by Bessis, in 1958 [76]. Erythroblastic islands consist of a central macrophage, surrounded by physically attached erythroblasts, which mature from proerythroblasts via basophilic and polychromatophilic forms to orthochromatic erythroblasts, losing all organelles in the process [70,71,73]. Orthochromatic erythroblasts finally exit their cell cycle and enucleate, separating into a reticulocyte and into a pyrenocyte [70,71,72]. Reticulocytes contain most of the cytoplasm [77] and enter into the bloodstream, where they mature within a time frame of approximately two days [71]. Pyrenocytes consist of the nucleus which is surrounded by a thin rim of cytoplasm and the plasma membrane [78]. They are readily phagocytized by the central macrophage [70,71,73], which is promoted by phosphatidylserine residues on their surface, acting as “eat-me” signals [71,72,79]. Engulfed nuclei are subsequently digested by DNaseII in lysosomes [72,80].

Although, in the literature, erythroblast enucleation has been repeatedly suggested to be a potential source of cfDNA [27,81,82,83], experimental evidence for this hypothesis is relatively sparse. When analyzing blood plasma of sex-mismatched bone marrow transplantation patients via quantitative real-time PCR (qPCR), Lui et al. (2002) [84] found that plasma cfDNA was predominantly of donor origin, and they concluded that cfDNA stemmed largely from cells of the hematopoietic lineage. This conclusion was confirmed by methylation-based tissue-mapping approaches [85,86] and by nucleosome footprint analyses [43], where it was stated that approximately 55% of cfDNA originated from white blood cells and 30% from erythrocyte progenitors [86,87]. Exercise-induced elevation of cfDNA levels in the blood was also attributed to cells of the hematopoietic linage by Tug et al. (2015) [83] in an analysis of cfDNA from sex-mismatched hematopoietic stem cell recipients via qPCR. Using high-resolution methylation profiles, Lam et al. (2017) [88] identified three genomic loci featuring erythroblast-specific low methylation density. They determined that erythroid DNA represented about 30% of the plasma cfDNA via qPCR and postulated that degraded erythroblast DNA from bone marrow could somehow escape into circulation. The question of how exactly DNA from pyrenocytes relates to cfDNA fragments, which can be found in the blood plasma or serum, remains largely unanswered thus far.

## 4. NETosis

Another mechanism, the release of neutrophil extracellular traps (NETs), which can lead to the creation of cfDNA, was discovered more recently [89]. NETs have been characterized as an innate response of neutrophils, with the function to trap and kill microorganisms. According to Yipp et al. [90], the indications for this process, which have been termed NETosis [91,92], were observed earlier, but not named or recognized as an independent event. The NETosis process is now accepted as an independent reaction to a threat and is considered to be different from apoptosis and necrosis.

NETs consist of disintegrated chromatin, which “trap” the microorganisms, and also serves as an anionic binding matrix for different antimicrobial proteins in order to kill the entrapped bacteria [90,93,94]. The proteins are usually found in granules inside of the cell and are released during NETosis. This release can occur either slowly, via a lytic cell death, or rapidly, via an explosive discharge of disintegrated chromatin and peptides, while the cell remains functional and can still respond to the surroundings [93,94]. These two different forms of NETosis are termed suicidal and vital [90], respectively. The mechanism of the NET formation was initially considered to be solely the result of NADPH oxidase-dependent production of reactive oxygen species (ROS) [91], but evidence for NADPH independent pathways was discovered subsequently [95,96]. There was also evidence that cell cycle proteins could be regulating NET formation [97].

Persistent NET structures in the bloodstream can lead to vascular occlusion [40], therefore, an effective removal system is required. It has been shown that the degradation of the released chromatin starts by host DNases circulating in the body fluid. The degradation is performed by two independent DNases, namely, DNaseI and DNaseI L-3 [40]. This breakdown of the chromatin is not the only process of NET removal, as the full clearing is performed with the support of macrophages [98]. It seems that DNases are involved with preprocessing the NET structure, while the total NET removal is performed by macrophages and other cell types incorporating and digesting the remainders of the NETs.

There is an indication that NETs may have an influence on cfDNA levels, since large amounts of DNA are released into the bloodstream, or into tissues, in a relatively short time. This influence has been studied for different health conditions and can correspond to increased cfDNA levels in diseased patients [99]. The cfDNA levels are also influenced by stalling the degradation process. Several diseases have been shown to lead to problems with a regulated full degradation of NETs, thus leading to higher cfDNA levels [100,101,102,103].

In addition to the degradation by endogenous DNases, it has also been shown that different species of bacteria could escape NETs by degrading the chromatin matrix via the release of DNases [104,105,106,107]. It may be possible that bacterial DNases produce different fragments of NETs as compared with the endogenous DNase degradation system, which could lead to an altered degradation pattern. To date, there is a lack of evidence for all of these possibilities. To protect the NET structures and preserve their antimicrobial character, the immune system coats the chromatin fraction with peptides, such as LL-37 and defensin-3 against degradation, which then hinders the activity of these DNases [108].

NETs do not always have beneficial effects during an immune response and the response to a threat by NETosis can even have detrimental effects for the organism. It has been shown, for example, that dormant cancer cells can be reactivated during inflammation reactions involving NETs in mice [109]. In chronic inflammation reactions, the lytic cell death mechanism has been shown to be proinflammatory, and therefore could counteract medical treatments [110]. It was also shown by Katkar et al. [111] that an exaggerated immune response, containing NETs together with the lack of DNase activity, was the main cause of tissue destruction by snake venom in mice. Tissue destruction due to NETosis and the following immune response were shown in this particular response, and also in other instances [99]. The same response pattern, together with increased cfDNA levels, has also been shown in patients with active COVID-19 infection, as recently reported by Zuo et al. [112].

## 5. Macromolecular Structures

Gahan and Stroun [113] coined the term virtosome, in 2010, to describe a circulating DNA-RNA-lipoprotein complex, which was actively released from living cells. Active release of DNA was first observed for stimulated lymphocyte cells [114]. Subsequently, DNA release was also shown for non-stimulated rat and human lymphocytes [115,116]. Furthermore, DNA release was observed for different eukaryotic cell types, for example, frog heart auricles [117] or rat spleen [118] mostly using in vitro studies, but it was also observed in vivo, for example using chick embryo fibroblasts [119]. The release was dependent on the concentration of the complex in the medium, with high concentrations in the environment suppressing further release [115,120]. The release mechanism itself was mostly unknown, however it was suggested that it may appear in phase G0 or G1 of the cell cycle [113].

It has been confirmed previously, by means of a ³H-thymidine labeling study, that DNA was newly synthesized within the cell [115]. The presence of proteins and lipids after treatment of the complex with proteinase K or lipase was observed and showed RNase activity [118]. A recent study by Cataldi and Viola-Magni [120] quantified the composition of virtosomes released from human lymphocytes into both the cytoplasm and the cell culture supernatant. This study found that the complexes in the cytoplasm contained approximately 3.45% DNA, 35.09% RNA, 19.90% phospholipids, and 41.01% proteins. Correspondingly, the cell culture supernatants contained 3.92% DNA, 36.41% RNA, 32.21% phospholipids, and 27.44% proteins. It was also suggested that there was no membrane around the virtosome [121], which was further supported by the low proportion of cholesterol and phosphatidylcholine in the complex [120]. These DNA molecules were believed to be around 450–700 bp long [113]. To our knowledge, there have not yet been any reports describing sequencing results (DNA and RNA) of virtosome complexes, which could be used to shed light onto the composition and specific origin of this fraction of cfDNA. Cells can uptake the virtosome complex [113] that has been released from cells of different cell types and the virtosomes can modify the biology of the receiving dividing cells [122,123]. This indicates that the virtosome complexes may be involved in signaling pathways between different cell types or horizontal gene transfer.

It is worth noting that Gahan and Stroun [113] pointed out several similarities between virtosomal DNA and metabolic DNA. It has been suggested that this unique population of DNA results from unscheduled DNA synthesis and occurs independently from the high molecular weight chromatin mass in cell nuclei [27,124]. In an early study of adult mouse heart, intestine, and skeletal muscle, it was found that metabolic DNA ranged between 500,000 and 4,000,000 Daltons [125] which equated to approximately 769 bp–6 Kbp in size. Interestingly, cfDNA fragments which exhibited this size profile have been observed in the supernatant of various cultured cell lines [126,127,128] and have been shown to be part of the cargo of some EVs [62,129,130]. They are often encountered in human biospecimens [131,132,133]. While it is an interesting possibility that metabolic DNA could serve as the precursor to larger ~1–6 Kbp cfDNA fragments, more research is needed to determine whether they truly represent a population of cfDNA originating from a regulated extrusion pathway, or whether they are simply the product of passive release mechanisms, such as apoptosis or necrosis.

## 6. Extracellular Vesicles

**Figure 2:**
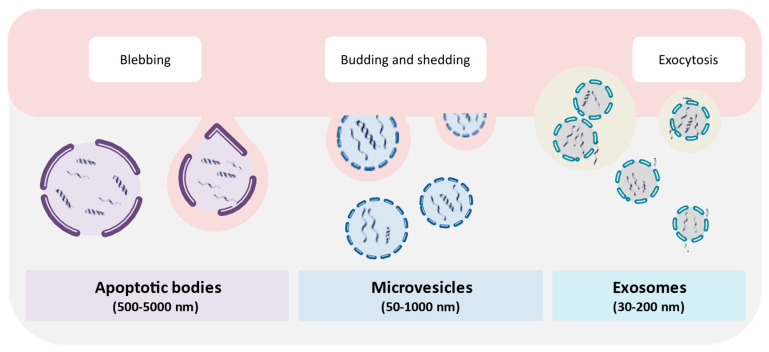
Different extracellular vesicle (EV) types in the human body. The three main types of EVs that occur in human body fluids include microvesicles, apoptotic bodies, and exosomes. These vesicles can be differentiated based on the mode of their production, cellular extrusion pathways, and their overall size. In addition, EVs can be stratified according to their content and function.

The release of EVs by cells was first described in 1967 for chondrocytes [134] and blood platelets [135]. These vesicles are characterized as single-lipid bilayer membrane extracellular organelles of simple spheroid morphology, which are present in all biological fluids tested thus far [136,137,138]. Constitutive release of EVs was reported for prokaryote [139] and eukaryote [134,135,136,137,138] cells and was considered to be a part of the normal physiology of all cells [138,140]. Currently, three main types can be distinguished, based on their size and their mechanisms of biogenesis [136,137,141] (Figure 2). Apoptotic bodies with diameters of 500–5000 nm are formed in the course of cell disintegration during programmed cell death [53,142]. Ectosomes, which encompass microparticles, microvesicles, and large vesicles with diameters from 50 to 1000 nm, are released directly from the plasma membrane via outward budding and fission [142,143]. Exosomes, with diameters of 30–200 nm, have been released via fusion of multivesicular bodies (MVBs) with the plasma membrane [136,137,141,143], which was first shown for rat reticulocytes, in 1983 [144]. The association of EVs with dsDNA was initially reported for human prostasomes, which were EVs secreted by the tissue of the prostatic gland, in a preliminary lab report from Olsson and Ronquist, in 1990 [145]. This result was finally confirmed almost twenty years later by Ronquist et al. (2009) [146]. In the following year, Guescini et al. (2010) [147], reported the presence of mitochondrial DNA (mtDNA) in EVs, which were characterized as exosomes released from astrocytes and glioblastoma cells. In prokaryotes, the release of DNA-containing outer membrane vesicles (OMVs) was first described, in 1989, by Dorward et al. [148] for *Neisseria gonorrhoeae* and by Garon et al. [149] for *Borrelia burgdorferi*. The studies also both showed that the encapsulated DNA was protected from digestion by DNaseI. When screening for viruses in hyperthermophilic archaea of the order Thermococcales, Soler et al. (2008) [150] observed the release of spherical membrane vesicles containing cellular DNA, which were not related to any viral activity and also exhibited resistance to DNase digestion.

A number of studies have demonstrated that DNA was affiliated with exosomes [129,151,152,153] and microvesicles [154,155], which were, for example, derived from diverse tumor types [129,151,152,154,156] or normal cells [155,156,157,158]. Furthermore, it has been shown that DNA molecules originating from both healthy [155,157,158] and tumor [153,154,159] cells could be transferred to other cells via EVs and may affect gene expression [155,157,158,159]. Similar observations have been made for bacterial DNA shuttled by OMVs [148,160]. Apart from affecting the gene expression of recipient cells, stable genomic integration of DNA transferred by vesicles was also shown in a proof-of-principle experiment by Fischer et al. (2016) [161]. Therefore, it is clear that EVs constitute a distinct, and perhaps biologically active, subpopulation of cfDNA molecules. However, several aspects concerning EV-associated cfDNA remain poorly understood, mainly because the phenomenon has, thus far, been underinvestigated and conflicting results have been reported in the literature.

One reason is that a fraction of the total cfDNA population in the blood plasma or serum, which is associated with EVs has not yet been clearly defined. An early study suggested that, rather than floating freely, the majority of cfDNA in the blood of cancer patients was contained within exosomes [152]. In support of this, Fernando et al. (2017) observed that up to 90% of the cfDNA in human blood was exosome-associated [62]. In contrast, Helmig et al. (2015) found that only about 5% of the total cfDNA population in blood collected from healthy individuals before and after physical exercise was associated with EVs [82]. Taking into consideration the relatively short half-life of free-floating cfDNA [87,162], the discrepancy between the reported proportions of vesicle-associated cfDNA in the above studies may be explained by different sample handling procedures. For example, in the study by Helmig et al., 2015, [82], the blood was analyzed directly after the blood draw, while the blood samples used in both the Kahlert et al. (2014) [152] and Fernando et al. (2017) [62] studies were taken at an external facility with a larger temporal delay before the analysis.

A second aspect is the question that, prior to its release from cells, it is not clear what fraction of the genomic DNA is packaged within the interior of the EVs vs. molecules that are attached to their exterior surface. In line with this, it is not known how much cfDNA can and does attach to different EVs after entering the extracellular space. In the above study by Helmig et al. (2015) [82], DNase treatment suggested that only about a quarter of the EV-associated DNA was contained within the vesicles. This was consistent with the findings of Fischer et al. (2016) [161] and Lazaro-Ibañez et al. (2019) [163] who reported that cfDNA was mainly associated with the outer membrane surface of EVs, with a smaller amount being contained within the vesicles. However, additional research is needed to determine the exact proportions in baseline conditions, as well as changes upon various stimuli.

Third, relatively little is known about the biological and physiological characteristics of EV-associated DNA, including, but not limited to the following: (i) the mechanisms that underlie the vesicle-mediated release of DNA molecules, (ii) the relative proportion of the total EV-associated cfDNA population that is complexed with each of the different EV types (i.e., apoptotic bodies, microvesicles, and exosomes), (iii) the physiological factors that modulate these mechanisms, and ultimately (iv) the relative contribution of different cell types toward the total EV-associated cfDNA population. Despite the lack of research on these topics, some recent studies provide at least some insights. Takahashi et al. (2017) [164], described the sustained exocytosis of DNA fragments via exosomes as an important physiological process for the preservation of cellular homeostasis, where the inhibition of exosome secretion led to intracellular accumulation of cytosolic DNA. This resulted in the activation of the ROS-dependent DNA damage response, followed by senescence and ultimately cell death. They also observed an increased release of DNA via exosomes in senescent cells. This suggests that cytosolic DNA fragments can be a significant source of intracellular stress, which is connected to increased exosome secretion, as described by Hessvik and Llorente (2017) [136]. MVBs can either be directed to lysosomes, where their content is degraded, or to the cell membrane, where they fuse with the membrane and release their vesicular content into the extracellular space as exosomes [136,142]. The destination of MVBs is considered to be dependent on cellular homeostasis [136,165,166]. Such an increase in exosome secretion in connection to a stress response has also been described for bacteria [160]. It is also noteworthy that Brahmer et al. (2019) [167] reported an increased release of EVs triggered by exercise. They identified endothelial cells, platelets, as well as different types of leukocytes, to be the main sources of these vesicles. Since the DNA cargo of the vesicles was not addressed in the study, the question of whether the increase in vesicles could lead to an increased amount of vesicle-associated DNA in the bloodstream remains currently unanswered.

Fourth, and in line with the previous point, the composition and size of the cfDNA molecules that are associated with EVs is unclear. Studies have shown that EVs contain fragments of single-stranded DNA [154], double-stranded DNA [129,151,152], as well as mtDNA [147,153]. These fragments have been found to range from as small as ~100 bp up to sizes exceeding 10 Kbp [129,152,154]. While the excreted cytosolic DNA fragments have long been thought to be a product of DNA damage [159,164], the heterogeneous composition of the exosomal DNA content, which features disproportionally large amounts of DNA from retro-transposable elements (RTEs) and satellite repeat DNA [154,168], suggests that other mechanisms could contribute to the abundance of cytosolic DNA fragments in a significant way. This overrepresentation of DNA from RTEs and satellite repeats has been found for cancer-cell culture supernatants [168,169], as well as the serum of healthy human subjects [169]. The respective fractions of these elements were significantly increased in human sepsis patients (bacterial and fungal sepsis), indicating an increased release in response to their medical condition [169].

## 7. Chromosomal Instability and Micronucleation

Recent reports have indicated the possibility that a portion of the total cfDNA population in vitro, as well as in vivo, could be a product of the effects of chromosome instability (CIN). CIN is one form of genetic instability that is characterized by sustained and often accelerated changes in the chromosome structure or number [170]. There are different pathways through which CIN results in the release of genomic DNA into the extracellular space, each of which can be modulated by a wide range of biological factors, thus, representing multiple layers of regulation [168]. Here, we limit the focus to specific chromosome mis-segregation events that can occur during mitosis.

### 7.1. Fundamental Chromosome Segregation Errors During Mitosis

After DNA replication, chromosomes possess two kinetochores, which are complex protein structures that serve as attachment points for the mitotic spindle. In the prometaphase, these kinetochores interact with spindle microtubules in both a lateral and end-on fashion. During the metaphase, each kinetochore binds to microtubules oriented towards opposite spindle poles. These bi-oriented kinetochore-microtubule (k-MT) attachments are crucial for the proper alignment and segregation of the chromosomes that are attached to the spindle [171]. However, due to the asynchronous and stochastic nature of the initial capture of microtubules by kinetochores, some k-MT attachments are not bi-oriented, resulting in either delayed or inappropriate attachment of chromosomes to the spindle during the prometaphase-to-metaphase transition [172]. Among the various k-MT attachment errors that can occur, merotelic attachments constitute a pathway for the generation of cfDNA. In merotelic attachments, one kinetochore binds to microtubules growing from opposite spindle poles [173,174]. Since merotelic attachments are not detected by the mitotic spindle checkpoint, cells can proceed to anaphase without completing error correction [175]. Although most merotelic chromosomes segregate correctly during anaphase, a small fraction remain at the spindle equator, resulting in lagging chromosomes [173]. Interestingly, if there is sufficient distance between a lagging chromosome and the main chromatin mass at the end of cell division, the lagging chromosome can recruit its own nuclear envelope and form a so-called micronucleus (MN), which is also known as a Howell–Jolly body [176]. In such cases, an interphase daughter cell contains the following two types of nuclei: (a) the primary large nucleus and (b) up to several smaller micronuclei (MNi) that house the mis-segregated chromosomes. While chromosome lagging and micronucleation can occur at low levels in normal cells [175,177], increased levels of MNi have been shown for various pathologies, especially in cancer cells with CIN [178,179,180,181,182]. In keeping with this, we hypothesize that MNi, with their DNA cargo, may translocate to the extracellular space and serve as one of the sources of cfDNA.

### 7.2. Other Chromosome Mis-Segregation Events that Can Arise During Mitosis

Apart from fundamental chromosome segregation errors, such as merotely, MNi can also arise during mitosis as a result of telomere loss. Telomeres preserve genomic stability by protecting natural chromosome ends from degradation, illegitimate self-recombination, and end joining with nearby chromosomes [183]. However, in certain cases (e.g., cancer), accelerated shortening of telomeres (loss of the terminal sequence), or complete loss of the end-capping structure is a prevalent feature [168,184,185,186,187]. Loss of telomeres is mainly the consequence of the following: (i) attrition of telomere repeats, which is typically associated with repression or reduced activity of telomerase [188]; (ii) loss of specific telomeric proteins, which may prompt the cell to identify chromosome ends as DNA breaks [189]; or (iii) double-stranded DNA breaks (DSBs), which may form as a result of hypomethylation followed by transposon-induced breaks [190,191,192], mis-repaired breaks caused by radiation [193,194,195,196], and deprivation of important metabolites, for example, folate [197,198]. When a telomere-deficient chromosome is replicated, the two ends of the sister chromatids fuse and form a chromosome with two centromeres, i.e., a dicentric chromosome [199,200]. Since a dicentric chromosome is able to attach to both spindle poles, the two centromeres are pulled to opposite poles during the anaphase, forming a continuous string of chromatin, stretching from one pole to the other, normally referred to as an anaphase bridge [201]. Anaphase bridges often break, resulting in various chromosomal abnormalities, often followed by the formation of MNi. The characteristics of the MNi and its DNA content depend on the location of the breaking point in the anaphase bridge [202,203,204,205].

**Figure 3:**
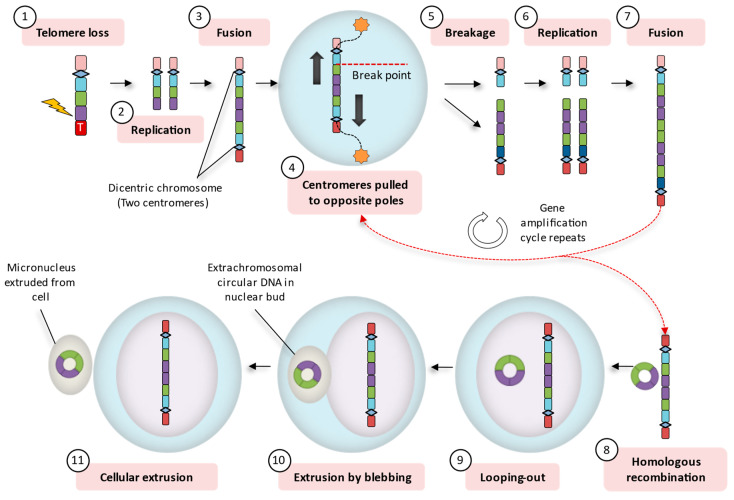
Breakage-fusion-bridge cycles as a potential source of cell-free DNA (cfDNA). A chromosome that has lost its telomere (**1**) is replicated (**2**) and the sister chromatids fuse at the broken ends, forming a dicentric chromosome (**3**). The two centromeres are, then, pulled to opposite spindle poles during the anaphase, stretching out the chromatin to form an anaphase bridge (**4**). In many cases an anaphase bridge breaks (**5**) and the broken telomere-deficient chromosomes are again replicated (**6**) and once again fuse with their sister chromatids (**7**). Several repetitions of this process result in the amplification of DNA sequences that are adjacent to the break or fusion point. An anaphase bridge may break in various regions, resulting in the amplification of different genomic regions (e.g., genes, oncogenes, and repetitive DNA). Recombination between homotypic sequences within the amplified DNA generates extrachromosomal circular DNA (**8**), which can eventually become looped out of the chromosome (**9**). These mini circles of DNA can subsequently be extruded from the nucleus through budding (**10**) and eventually become extruded from cells into the extracellular space (**11**).

Repeated pulling apart of dicentric chromosomes, anaphase bridge formation, and subsequent breakage in gene regions over multiple cell divisions, also known as breakage-fusion-bridge (BFB) cycles, results in the amplification of DNA sequences that are adjacent to the break or fusion point [201,206]. The BFB cycles are typically sustained until the chromosome acquires a new telomere. However, during the process, recombination of homotypic sequences within the amplified DNA often results in the formation of mini circles of acentric and atelomeric DNA, which are eliminated from the aberrant chromosome. These structures, which are known as double minutes (DMs) [201,206], are capable of replication and can also localize to the nuclear periphery, exit the nucleus through budding, and eventually become extruded from cells in the form of microcells [203,207]. When they are in the form of nuclear buds (NBUDs) after exiting the nucleus, they have a similar morphology as the typical MN [202]. Interestingly, this extra-chromosomally amplified DNA frequently consists of oncogenes [208,209,210]. This process, which is summarized in Figure 3, may explain the recently reported presence of extrachromosomal circular DNA in the circulatory system of both mice and humans [211,212,213]. It may also explain the significant overrepresentation of specific retrotransposons in the cfDNA isolated from the cell culture supernatant of human bone osteosarcoma (143B) cells [168].

Anaphase bridges can also break in non-gene regions. Differentiation between chromosomes with varying telomere erosion profiles showed that the long and short arms of chromosomes 1 and 22, respectively, had critically short telomeres as compared with other chromosomes. Consistent with this observation, telomeric fusion of these chromosomes was the most striking cytogenetic abnormality observed at anaphase after the first population doubling of human mammary epithelial cells. However, since chromosome 1 contributed more to the formation of anaphase bridges, it was suggested that the loss of telomere function did not modulate the formation of nuclear anomalies through a single mechanism [214]. This concurred with a previous study showing that telomere dysfunction caused a spectrum of mitotic defects [194]. In this study, two different types of anaphase bridges were observed, i.e., one type consisting of one or two strings of decondensed chromatin connected to both spindle poles, whereas the other bridge had detached from either one or both spindle poles (resulting in lagging of entire chromosomes). Fragmented DNA was localized either in NBUDs, which contained completely fragmented and broken anaphase bridges, or in internuclear strings, which originated from anaphase bridges that were fragmented, but not fully broken. Interestingly, in more than 90% of strings and protrusions, DNA fragmentation began adjacent to the centromere and continued distally. Karyotype analysis of 338 cases of colorectal adenocarcinomas confirmed that this pericentromeric breakpoint pattern also occurred in vivo [194].

In addition to the above observations, molecular studies of immunodeficiency, centromeric region instability, and facial anomalies (ICF syndrome) have shown that chromosomes 1 and 16 and, in some cases, chromosomes 2 and 9 were predisposed to hypomethylation at their centromeric and pericentromeric regions [215], resulting in a variety of cytogenetic abnormalities, which included the extrusion of self-associated satellite DNA into MNi or NBUDs [216]. In line with this, several cancer types have shown preferential hypomethylation of the satellite repeat arrays on the basis of the q-arms of chromosomes 1, 9, and 16 [217,218,219]. This hypomethylation was causally linked to a high frequency of non-random rearrangements in the centromeric and pericentromeric heterochromatin of these chromosomes, which were nearly identical to those observed in ICF syndrome. In support of this, similar genomic aberrations and MNi-containing DNA derived from the centromeric and pericentromeric regions of chromosomes 1, 9, and 16 were induced in cultured cells through exposure to various clastogenic compounds that prevent normal methylation [220,221,222,223,224]. Taken together, these studies may explain why a growing number of studies have reported an overrepresentation of repetitive DNA in cfDNA [168,169,225,226,227]. More specifically, these studies may provide an explanation for the question why cfDNA derived from cultured cancer cells has been found to be enriched in repetitive DNA, which originated from the centromeric and pericentromeric regions of chromosomes 1, 9, and 22 [168].

While these studies provided indirect evidence that a portion of the cfDNA originate from CIN-induced MNi, there is currently no direct experimental evidence that shows this. Indeed, as far as we know, there have been, thus far, no attempts to isolate “free-floating” MNi directly from human body fluids. Therefore, further studies on the nature of MNi would likely provide deeper insight into the biology and physicochemical properties of cfDNA.

## 8. Discussion

Cell death, primarily via apoptosis or, under certain circumstances, necrosis, has often been considered to be the only relevant mechanism of DNA release into the bloodstream. This assumption has been justified by the non-random fragmentation pattern of circulating cfDNA [44,66,67]. In contrast, several active mechanisms for the release of cfDNA from cells have now also been described, where, for instance, a considerable fraction of the total cfDNA, which is attributed to the erythroid cell lineage [85,86,87,228], cannot sufficiently be explained by cell death, since mature erythrocytes do not possess a nucleus when undergoing eryptosis (erythrocyte apoptosis) [229]. Additionally, the mechanism of NETosis, which contributes substantially to the cfDNA fraction is not considered to be a passive mechanism [92,230]. Active release of DNA has been observed in many parts of the eukaryotic kingdom, i.e., it has been observed, for example, in humans, rats [231], chickens [232] and *allomyces arbusculus* (fungi) [233]. However, in these papers, there were no detailed characterizations described which dealt with the mechanism of cfDNA or the associated functions. This led Elzanowska et al. (in 2020) [234] to conclude that ”DNA can also be released from living cells by active cellular secretion, although at present little is known about why functional cells secrete DNA and what is the biological significance of this process.

An entirely random release of DNA into the bloodstream as the predominant mechanism of cfDNA generation (i.e., essentially resulting fragmented entire chromosomes entering the bloodstream) contradicts, in our opinion, the fact that exosomal cfDNA clearly has an effect on cultured cells. For example, when exosomes hailing from radiated cell cultures were inoculated into cell cultures, which were not radiated, this led to a similar phenotype in the non-radiated cells as in the radiated cells [235,236,237,238]. In addition, the fact that exosomal cfDNA can be incorporated into the nuclei of inoculated cells [161] within a short time frame allows us to speculate that the cfDNA molecules contained in these exosomes may contain messages, which can be utilized in the uptaking cell.

Passively released cfDNA is still a useful tool, which can be used in a biomedical context for certain diseases, although, in our opinion, this is limited to diseases which are connected to dramatic changes to the genome of the patient (e.g., chromosomal rearrangements in tumors). Currently, only a small number of diagnostic tests using cfDNA are approved by the U.S. Food and Drug Administration, for example, the cobas^®^ EGFR Mutation PCR Test v2 for non-small cell lung cancer [239], a test for ctDNA for PIK3CA mutated hormone receptor positive breast cancer [240], and the SEPT9 methylated DNA test for colorectal cancer [241]. The most prominent example is certainly the prenatal diagnostic test for Down syndrome and other chromosomal abnormalities [23,24,25,26], which is, to our knowledge, currently the only widely used cfDNA based diagnostic procedure available in public health institutions around the globe. Several groups are also developing screening tools for resistance in cancers, using cfDNA fragments originating from tumor tissue and constructing maps of tumor chromosomes after high-throughput DNA sequencing to determine mutations (insertions/deletions) on the cancer chromosomes [242,243,244]. As the detection limits for this cfDNA fraction are lowered (currently they lie around 2–5% of the total cfDNA), this is becoming a very valuable tool for non-invasive diagnosis of cancers [66,245].

Elevated levels of circulating cfDNA have been reported for several forms of physiological stress such as different disease conditions [12,13,15,99,246] or even simply exhaustion from physical exercise [82,83,247]. Correspondingly, increased active release of DNA fragments from cells via exosomes was found in connection with intracellular stress and senescence [164]. It is, in our opinion, unlikely that these cfDNA fractions are solely the product of apoptosis and necrosis, as very often the increase in cfDNA levels in the bloodstream occurs shortly after the stress exposure and is quite substantial [162]. The interplay of multiple cfDNA release mechanisms makes it difficult to relate the profile of cfDNA in blood to distinct diseases. We have experienced this ourselves when we investigated the occurrence of certain cfDNA sequence motifs (biomarkers) in relation to postsurgical bacteremia and sepsis [248]. Evaluating the frequencies of the motifs relative to the total cfDNA concentration of the samples did not yield any reliable correlation to the disease state. Therefore, we had to assume that several mechanisms contributed to the total cfDNA population being also overlaid (and thus partially masked) by the response to non-disease-related physiological causes, thus, interfering with our attempts to normalize the disease signals. 

Evaluating the motif frequencies relative to each other in the form of motif pairs provided an intrinsic reference system, which ultimately enabled a motif-based distinction between samples from sepsis patients and samples from healthy probands [248]. These results provided evidence that information may be contained in cfDNA fragment frequency, methylation patterns, or the occurrence of mutations in some genomic regions, and also in the relative abundance of particular cfDNA motifs. The cfDNA fraction comprised by certain sequence elements, especially RTE and satellite repeats, seemed to change in response to physiological conditions [169,248], which could provide novel perspectives for future diagnostic approaches. Similar overrepresentation of these repeat elements has also been found in human bone osteosarcoma cancer cell culture supernatants [168]. Since different methods for DNA purification, amplification, and sequencing were used in these studies, it is very unlikely that these results were obtained due to artifacts caused by the applied methodology. Assessing the contribution of different cfDNA release mechanisms under varying physiological conditions may be crucial for the identification of additional reliable and robust signals within the information continuum provided by the cfDNA composition in body fluids. We expect that this should lead to the development of further diagnostic assays in the future. This research and development field is only emerging now, with very detailed studies possible due to third-generation high-throughput DNA sequencing methods being applied to the characterization of the entire DNA content of serum or plasma samples from patients and controls. Unlike the diagnostic assays, which solely consider passively released DNA as the target, the efforts focusing on including actively released cfDNA molecules have the advantage that they deal with the full spectrum of DNA circulating in the bloodstream, thus, increasing the signal-to-noise ratio considerably.
